# Epidemiological and molecular study of hemoglobinopathies in Mauritanian patients

**DOI:** 10.1002/mgg3.2048

**Published:** 2022-09-15

**Authors:** Taher Mahmoud, Chaima Sahli, Sondess Hadj Fredj, Yessine Amri, Rim Othmani, Ghaber S. Mohamed, Ekhtelbenina Zein, Taieb Messaoud

**Affiliations:** ^1^ Biochemistry and Molecular Laboratory LR00SP03 Children's Hospital of Tunis Tunis Tunisia; ^2^ Medical Analysis Laboratory (MauriLab) Nouakchott Mauritania; ^3^ Doctoral School (STVST) of the Faculty of Sciences of Tunis University of Tunis El Manar Tunis Tunisia; ^4^ Pediatric Hemato‐Oncology Department of the National Oncology Center (CNO) Nouakchott Mauritania

**Keywords:** haplotypes, hemoglobin (Hb) disorders, hemoglobinopathies, sickle cell disease (SCD), α‐thalassemia, β‐thalassemia

## Abstract

**Background:**

Hemoglobinopathies, inherited disorders of hemoglobin (Hb), are the most common hereditary monogenic diseases of the red cell in the world. Few studies have been conducted on hemoglobinopathies in Mauritania. Therefore, the aim of this work is to establish the molecular and epidemiological basis of hemoglobinopathies in a cohort of Mauritanian patients and to determine the haplotype of the β‐globin gene cluster in sickle cell subjects.

**Methods:**

The molecular screening of Hb disorders in 40 Mauritanian patients was done by a polymerase‐restriction fragment length polymorphism (RFLP) for the sickle cell disease (SCD) mutation, a PCR/sequencing method for β‐thalassemia mutations, and by the multiplex polymerase chain reaction method for the α‐thalassemia. The exploration of eight polymorphic sites (SNPs) within the β‐globin gene cluster was conducted by PCR/RFLP method, to identify the HbS haplotypes from the sickle cell subjects.

**Results:**

The epidemiological study of our patients showed a high incidence in the Senegal River area (52.5%) and a high ethnic prevalence for the *Heratin* (47.5%) and the *Pular* (35%). Molecular study allowed us to identify eight different mutations in our sample analyzed. They are respectively: HbS (HBB:c.20A>T) (68.75%), Cd44 ‐C (HBB:c.135delC) (8.75%), −29A>G (HBB:c.‐79A>G) (4.8%), −α‐3.7 (g.34164_37967del3804) (3.75%), IVS‐II‐849A>G (HBB:c.316‐2A>G) (2.25%) and Cd24T>A (HBB:c.75T>A), Hb Siirt (HBB:c.83C>G) and HbC (HBB:c.19G>A) each with (1.25%). Six different haplotypes are being explored among the SCD subjects with the Senegal haplotype as the most prevalent (66.7%), followed by Benin (10%), Arab‐Indians (6.7%), Bantu (3.3%), and two atypical haplotypes.

**Conclusion:**

Our findings enrich the epidemiological data in our population and could contribute to the establishment of a strategy of prevention and management through screening, genetic counseling, and prenatal diagnosis of Hemoglobinopathies in the Mauritanian population.

## Graphical abstract

Hemoglobinopathies, inherited disorders of hemoglobin (Hb), are the most common hereditary monogenic diseases of the red cell in the world. Few studies have been conducted on Hemoglobinopathies in Mauritania. Our results showed a high prevalence of hemoglobinopathies patients in Bank Senegal River in the South‐West of the country. Two Mauritanian ethnic groups, the Heratin and the Pular, were the most affected.

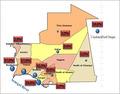

## 1 INTRODUCTION

Hemoglobinopathies are the most common hereditary monogenic diseases of the red blood cell in the world (Weatherall, 2007). They are a group of inherited disorders of hemoglobin (Hb) that are mostly transmitted in an autosomal recessive mode. These inherited disorders are characterized by either abnormal globin chain variants like sickle cell anemia or deficit, total or partial, globin chain synthesis in erythroid cells during hematopoiesis at the origin of thalassemia syndromes (Rees et al., 2010).

The most known forms are the β‐thalassemia which prevails primarily in the carrier of the Mediterranean basin, and the sickle cell disease (SCD) which is more frequent in the countries of sub‐Saharan Africa (Weatherall & Clegg, 2001).

The heterogeneous ethnic and epidemiological distribution of these diseases and the presence of wide variability in the clinical expression linked to the genetic and environmental factors constitute an obstacle for the development of hemoglobinopathies control programs (Zohreh, 2013).

The Mauritanian geographic situation constitutes a link between the population of West Africa and those from the Maghreb. The Mauritanian population consists of two big groups: the Arabo‐Berber whose members are commonly named Moor (Heratin and Bidan) and the origin African composed of Pular, Soninke, Wolof, and a minority of Bambara (Merchesin, 1992). Few studies have been carried out on hemoglobinopathies in Mauritania. The first study evaluated the frequency of the β^S^ at around 8.71% (Deyde et al., 2002), however, the second study shows a frequency of 5.71% (Veten et al., 2012).

With regard to the genetic study of Hemoglobinopathies, nearly a thousand mutant alleles have now been reported (https://globin.bx.psu.edu/hbvar/menu.html). The mutations are regionally specific and in most cases, the geographical and ethnic distributions have been determined providing the foundation for a program of control through screening, genetic counseling, and prenatal diagnosis (Giardine et al., 2021; Hardison et al., 2002; Swee Lay, 2013).

It should be noted that the HbS mutation has been described on five distinct haplotypes based on the presence or absence of the eight different restriction enzyme sites in the genomic regions retching from the 5′ε‐globin to the 3′β‐globin gene on chromosome 11 (~60 kb) (Kamel, 1979; Sutton et al., 1989). These haplotypes are known as Benin, Bantu or Central African Republic (CAR), Senegal, Cameroon, and Asian (Arabo‐Indian) according to their ethnic and geographical origins. In fact, the first four are African haplotypes (Pagnier et al., 1984), while the last one was described in Central India and in Saudi Arabia (Nagel & Fleming, 1992). It has been previously reported that the CAR haplotype is usually associated with a more severe disease when compared with the intermediate phenotype of the Benin haplotype and to the milder conditions associated with the Senegal and the Arabo‐Indian (Padmos et al., 1991). Analysis of the polymorphic sites of the β genes cluster is of genetic, anthropologic, and clinical interest, and it can also be used to predict disease prognosis and to plan appropriate treatment.

In this study, we aim to determine the molecular and epidemiological basis of hemoglobinopathies in a group of Mauritanian patients. To do this, we have explored the β‐ and α‐ globin genes to identify mutations responsible for hemoglobinopathies. Then, we focused on the exploration of eight polymorphic sites (SNPs) within the β‐globin gene cluster to establish the HbS haplotype in the sickle cell subjects. Also, the epidemiological study and the correlation phenotype/genotype study were carried out in order to better understand the clinical and hematological characteristics of hemoglobinopathies in our sample study.

## 2 MATERIALS AND METHODS

### 2.1 Patients

This study included 40 Mauritanian patients that were affected by hereditary hemoglobin diseases, mainly SCD, β‐thalassemia, and α‐thalassemia. They are originated from different Mauritania cities and are regularly followed in the Pediatric Hemato‐Oncology department of the National Oncology Center (CNO) and at the *MaurLab* laboratory in Nouakchott, Mauritania during 2018 to 2021. Information about blood transfusion, age of diagnosis, splenomegaly, vaso‐occlusive crises, and the hematological parameters were obtained by retrospective clinical data. Our subjects are aged between 1 and 29 years old with an average age of 10 years. Our cohort consists of 25 males (60.97%) and 15 females (39.02%) with 1.6 as gender ratio. Our demographic data indicates that 29.3% of patients were born from consanguineous couples.

After obtaining authorization, 3–5 ml of whole blood from each subject were collected on an EDTA tube and then referred to the Biochemistry and Molecular Laboratory at Children's Hospital of Tunisia for molecular study. Written consent was obtained from all patients and parents of minor patients before enrollment in the study.

This study was approved by the ethics committee of Pediatric Hemato‐Oncology department of the National Oncology Center in Mauritania and the Children's Hospital of Tunis.

### 2.2 Hematological data and hemoglobin analysis

Hematological parameters were determined by cytometry on an automated Beckman LH750 TM Hematology analyzer (Beckman). Hb identification was carried out by a cation exchange high‐performance liquid chromatography (HPLC), using Variant TM II System (Bio‐Rad Laboratories). Hb fractions were eluted from the cartridge based on their ionic interaction with the cartridge material, and the different Hbs were eluted in the order: HbF, glycated HbA, HbA, HbA 2, and HbS.

### 2.3 DNA analysis and molecular study

Genomic DNA was isolated from white blood cells by the salting‐out procedure (Miller et al., 1988) or by the PureLink® Genomic DNA Kit (Invitrogen, ThermoFiher Scientific). The research on β^S^ mutation [β6, GAG>GTG, Glu>Val] was carried out by the PCR‐RFLP (Polymerase‐Restriction Fragment Length Polymorphism) with the Bsu36I restriction enzyme (Livingstone, 1967).

Molecular screening of the β‐thalassemia mutations was done by PCR/Sequencing, using the specific primers (Chaima et al., 2013) and the SeqStudio™ Genetic Analyzer System (Thermo Fisher Scientific) for the systematic sequencing of β‐globin gene (*HBB* **)** (Sanger et al., 1977).

Common α‐thalassemia mutation –α^3.7^deletion was screened among patients, by multiplex polymerase chain reaction method (Arnold et al., 2001), using the Go Taq® Long Mater Mix kit (Promega).

The β‐globin gene cluster haplotype was characterized by the PCR‐RFLP method (Orkin et al., 1982; Saleh‐Gohari & Mohammadi‐Anaie, 2012). The common haplotype of the β‐globin gene cluster was determined according to the presence (+) or absence (−) of a composition of SNPs (Single Nucleotide Polymorphism) corresponding to the traditional eight restriction sites were analyzed including the *Hin*cII (5′ of ε‐globin gene), *Xmn*I (5′ of the Gγgene), *Hin*dIII (intron 2 of the Gγgene), *Hin*dIII (Aγ‐globingene), *Hin*cII (5′ of the ψβ‐globin gene), *Hin*cII (3′ of the ψβ‐globin gene), *Ava*II (intron 2 of β‐globin gene) and *Bam*HI (3′ of the β‐globin gene).

The statistical analysis was performed using version 20.0 of the statistical package for the social sciences software SPSS (SPSS). The SNPs analysis and the haplotypes determination were performed by the haploview 4.2 software.

## 3 RESULTS

**FIGURE 1:**
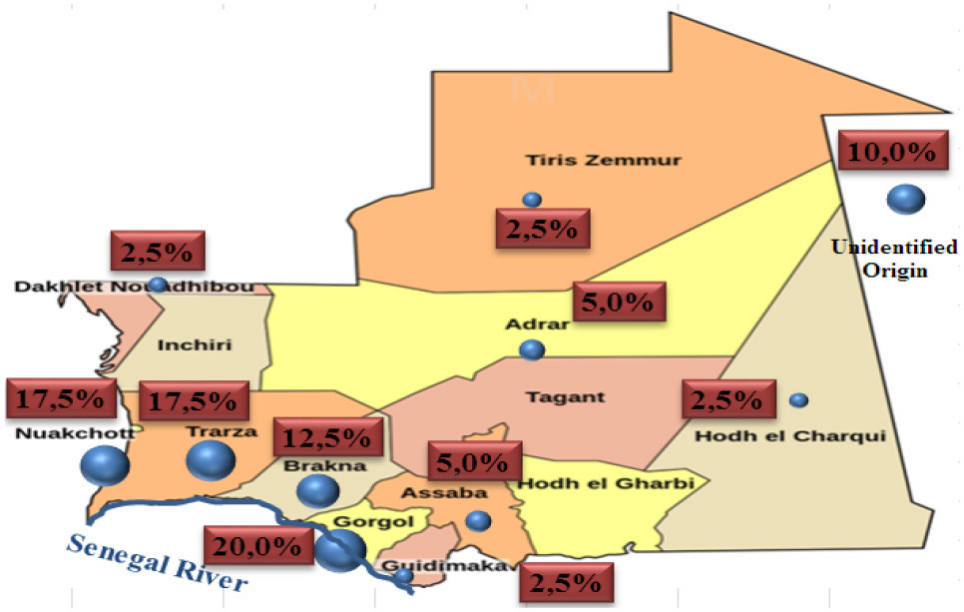
Geographical distribution of our patients in Mauritania

**FIGURE 2:**
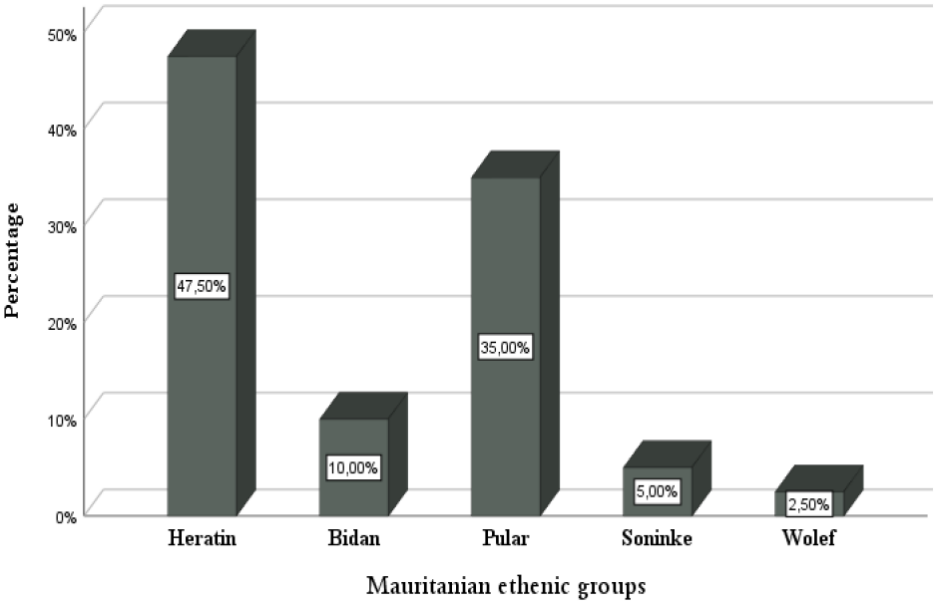
Ethnic distribution of our Mauritanian patients

The epidemiological data analysis showed a wide distribution of hemoglobinopathies throughout the Mauritanian territory with a high incidence on the area of the Senegal River bank (52.5%), located in the South‐West, which included the cities of *Gorgol*, *Guidimakha*, *Trarza*, and *Brakna*. The Mauritanian population is made up of two big groups: the Negro‐Africans (Pular, Wolof, and Soninke) and the Arabo‐Berbers (Heratin and Bidan). Indeed, the ethnic distribution of our sample showed a high prevalence of Hemoglobinopathies in favor of Heratin (47.5%) and Pular (35%) (Figures 1 and 2).

The molecular study allowed us to identify five types of Hb disorders (Hb) in overall samples: the HbS variant (β^S^) (c.20A>T) is the most frequent accounting 68, 75% followed by β‐Thal alleles (15%), α‐Thal alleles (3.75%), HbC (c.19G>A) (1.25%) and Hb Siirt (c.83C>G) (1.25%) (Table 1). It should be noted that the last variant is found for the first time in the Mauritanian population in a heterozygous state.

**TABLE 1:** Allelic frequency of hemoglobin defects found in our sample study

Hb defects	Number of alleles (%)
β^−S^	55 (68.75)
β^−Thal^	12 (15.0)
α^−Thal^	3 (3.75)
β^−A^	8 (10.0)
HbC	1 (1.25)
Hb Siirt	1 (1.25)

Eight different mutations were detected in our study (Table 2). The β‐thal point mutations were respectively the Cd44 ‐C (c.135delC) with 8.75%, the −29 A>G (c.‐79A>G) with 4.8%, the IVS‐II‐849 A>G (c.316‐2A>G) with 2.25% and the Cd24 T>A (c.75T>A) with 1.25%. –α‐3.7deletion (g.34164_37967del3804) was detected on the α‐globin gene with 3.75%. Three SNP were also reported: rs10768683 or IVS2‐16 C>G (15%), rs713040 or cd2 C>T (7.5%) and rs16098012 or IVS2‐666 (10%).

**TABLE 2:** Different mutations identified in our sample study and their ethnic origin (https://globin.bx.psu.edu/hbvar/menu.html)

Mutations	Type	Number of alleles (%)	Ethnic origin
HBB:c.20A>T	Hb Variant	55 (68.75)	Black American and Indian
HBB: c.135delC	β^0^	7 (8.75)	Oman, Tunisian, Bahraini…
HBB:c.‐79A>G	β^+^	4 (4.8)	Black & Chinese
NG_000006.1:g.34164_37967del3804	α^−thal−2^	3 (3.75)	Indian, Far Eastern; African, Mediterranean
HBB:c.316‐2A>G	β^0^	2 (2.25)	African black
HBB:c.75T>A	β^+^	1 (1.25)	American black, Japanese
HBB:c.83C>G	Hb variant	1 (1.25)	Kurdish
HBB:c.19G>A	Hb variant	1 (1,25)	African black
rs10768683 (G>C)	SNP	12 (15)	Indian
rs1609812 (C>T)	SNP	8 (10)	Indian
rs713040 (T>C)	SNP	6 (7.5)	Indian

Abbreviations: Hb variant, hemoglobin variant; β^+^, a partial deficit of β‐globin chains synthesis; β^0^, a total deficit of β‐globin chains synthesis; SNP, Single Nucleotide Polymorphism; α^−thal−2^, alpha‐thalassemia type 2.

The Genotypic analysis showed the presence of eight different genotypes in our subjects. The **β** ^**S**^ **/β** ^**S**^ (55%) and **β** ^**A**^ **/β** ^**−Thal**^ (17.5%) were the most common genotypes. While, the **β** ^**S**^ **/β** ^**C**^ was the less frequent one (Table 3). Moreover, our hematological data showed that the β^S^/β^thal^ genotype increases levels of hematocrite and HbA1, and decreases levels of HbF and HbS as compared to the **β** ^**S**^ **/β** ^**S**^. While, **β** ^**S**^ **/β** ^**S**^, **αα/−−** genotype decreases MCV, MCH, hematocryte, and HbA2 more than the **β** ^**S**^ **/β** ^**S**^ genotype (Table 3).

**TABLE 3:** The hematological parameters of the different genotypes identified in our study

Genotypes	Hb (g/dl)	MCH (pg)	MCV (fl)	Ht (%)	HbA (%)	HbA2 (%)	HbF (%)	HbS (%)	HbC (%)	*N* (%)
β^A^/β^S^	9.15	23.63	72.30	27.50	54.50	2.56	4.2	40.13		3 (7.5)
β^S^/β^S^	7.44	27.80	83.90	21.50	2.78	3.18	12.64	79.43		22 (55)
β^S^/β^C^	11.3	25	75	33	2.5	1.9	3.1	41.7	40.8	1 (2.5)
β^−Thal^/β^−Thal^	6.50	24.5	69.30	15.4	36.65	5.95	40.8			2 (5)
β^A^/β^−Thal^	9.38	21.23	74.4	12.33	70.08	5.51	18.00	0		7 (17.5)
β^S^/β^−Thal^	10.25	21.3	67.5	32.5	28.65	2.55	3.15	58.15		2 (5)
β^S^/β^S^, αα/−−	6.4	26.75	72.50	15	1.1	1.8	8.4	86.5		2(5)
β^A^/β^S^, αα/−−	10.30	19	61	33.0	47.10	1.2	0.70	33.80		1 (2.5)
β^A^/β^S^	9.15	23.63	72.30	27.50	54.50	2.56	4.2	40.13		3 (7.5)
β^S^/β^S^	7.44	27.80	83.90	21.50	2.78	3.18	12.64	79.43		22 (55)
β^S^/β^C^	11.3	25	75	33	2.5	1.9	3.1	41.7	40.8	1 (2.5)
β^−Thal^/β^−Thal^	6.50	24.5	69.30	15.4	36.65	5.95	40.8			2 (5)
β^A^/β^−Thal^	9.38	21.23	74.4	12.33	70.08	5.51	18.00	0		7 (17.5)
β^S^/β^−Thal^	10.25	21.3	67.5	32.5	28.65	2.55	3.15	58.15		2 (5)
β^S^/β^S^, αα/−−	6.4	26.75	72.50	15	1.1	1.8	8.4	86.5		2 (5)
β^A^/β^S^, αα/−−	10.30	19	61	33.0	47.10	1.2	0.70	33.80		1 (2,5)

Abbreviations: Hb, hemoglobin; MCH, mean corpuscular hemoglobin; MCV, mean cell volume; Ht, hematocrite; HbA, hemoglobin A; HbA2, hemoglobina2; HbF, fetal hemoglobin; HbS, sickle hemoglobin; HbC, hemoglobin C.

**FIGURE 3:**
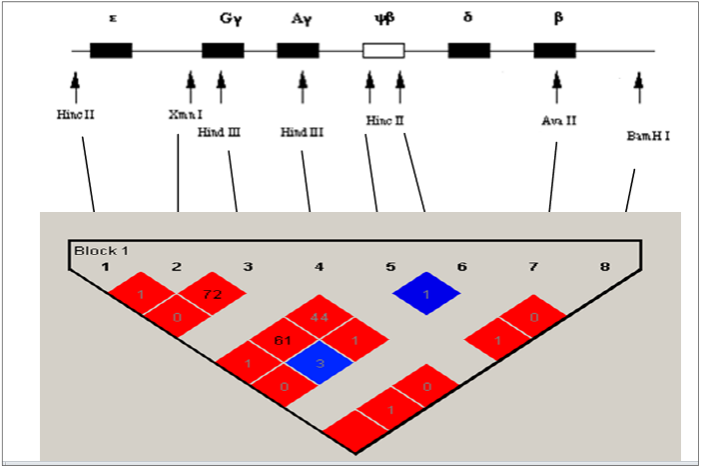
Different haplotypes found in our cohort

We were also carried out a molecular exploration of eight Single Nucleotide Polymorphisms (SNPs) within the β‐globin gene cluster in order to establish the haplotypes from 30 sickle cell subjects in our cohort. This study showed that there were six different haplotypes in our cohort, revealing the Senegalese haplotype as the most frequent (66.66%), followed by Benin (10%), Arabo‐Indian (6.66%), and Bantu (3.33%). In our cohort, we noted Two atypical haplotypes (different combinations), termed here as Aty1 and Aty2 with 6.66% each other (Table 4, Figure 3).

**TABLE 4:** Frequencies of restriction haplotypes established in sickle cell subjects

Haplotypes	3′‐ɛ Hinc II rs3834466	5′‐Gγ XmnI rs7482144	Gγ HindIII rs2070972	Aγ HindIII rs28440105	Ψβ HincII rs10128556	3′ ψβ HincII rs968857	β AvaII rs1076863	3′β BamHI no RSID	*n* (%)
Senegal	−	+	+	−	+	+	+	+	40 (66.7)
Benin	−	−	−	−	−	+	+	+	6 (10)
Arab‐Indian	+	+	+	−	+	+	+	−	4 (6.7)
Bantu	−	−	+	−	−	−	+	+	2 (3.3)
Atypical 1	−	+	+	−	+	−	+	+	4 (6.7)
Atypical 2	−	+	+	−	−	+	+	+	4 (6.7)

Abbreviations: +, presence of the SNP; −, absence of the SNP.

In order to understand the clinical heterogeneity observed from our SCD subjects, we have analyzed the clinical and hematological data related to each haplotypes. We noted that patients carrying the haplotype the Senegalese haplotype present a less severe phenotype compared to Benin, Bantu, and Arabo‐Indian haplotypes. In fact, among the subjects who carried the Senegalese haplotype only 6.66% present splenomegaly, 46.6% present a vaso‐occlusive crisis and 13.3% require a regular blood transfusion (Table 5).

**TABLE 5:** The clinical and hematological data associated with different haplotypes established from sickle cell subjects

Haplotypes	Hb (g/dl) *p* = 0.58	HbF (%) *p* = 0.35	HbS (%) *p* = 0.87	BT (%) *p* = 0.59	VOC (%) *p* = 1.00	SHM (%) *p* = 0.33
Senegal	7.47	11.09	74.82	13.3	46.6	6.6
Benin	7.36	9.15	71.31	33.3	50.0	33.3
Arabo‐Indian	10	6.8	69.05	50.0	50.0	50.0
Bantu	7.10	20.33	76.7	0.0	50.0	0.0
Atypical 1	8.10	5.45	56.35	0.0	50.0	0.0
Atypical 2	9.50	19.00	69.6	0.0	50.0	0.0

Abbreviations: Hb, mean Hemoglobin level; HbF, mean level of fetal hemoglobin; HbS, mean level of hemoglobin S; BT, blood transfusion frequency; VOC, vaso‐occlusive crises frequency; SHM, spleno‐hepato‐megaly frequency.

## 4 DISCUSSION

Hemoglobinopathies are widely spread throughout North Africa (Fattoum, 2009). They represent a public health problem in Mauritania due to lack of knowledge and diagnostic errors. In order to establish a molecular basis for these genetic disorders, a cohort of 40 Mauritanian patients was studied (Appendix A, Table A1).

The epidemiological data analysis showed an important distribution of hemoglobin disorders throughout the Mauritanian territory with a high incidence in the Senegal River bank (52.5%) located in the South‐West of the country. This high prevalence of hemoglobinopathies in the area of Senegal River bank could be due to speared of hemoglobin defects by the gene flow from Senegal to Mauritania, especially the SCD and α‐thalassemia, which have been reported as very common in the Senegalese population (Gueye Tall et al., 2017; OMS, 2006). The ethnic distribution in our group of patients showed a high incidence in favor of Heratin and Pular with 47.5% and 35% respectively (Figure 2). These results are in contradiction with those found by Deyde et al. (2002). This difference can be explained by the number of analyzed patients and the location of the hospitals study centers. Our finding indicates that these two Mauritanian groups ethnic (Heratin and Pular) may constitute a high‐risk population which implies the establishment of systematic screening program among their members.

Concerning the molecular screening of α‐ and β‐globin genes, we detected eight different mutations and three SNPs in the 82 alleles studied. In fact, the β^S^ mutation, was found to be the most common with 68.75%, followed by Cd44 ‐C (c.135delC) (8.75%), −29 A>G (c.‐79A>G) (4.8%), −α‐3.7 (g.34164_37967del3804) (3.75%), and IVS‐II‐849 A>G (c.316‐2A>G) (2.25%) mutation.

Most of these mutations have been reported previously in the African, Mediterranean, and Asian population (Amselem et al., 1988; Antonarakis & Cheng, 1984; Schilirò et al., 1995). This finding is in line with the history of the Mauritanian population that was a link between the Black population of West Africa and those from the Maghreb (Merchesin, 1992).

The Hb Siirt variant was found in a 16‐year‐old patient who presented clinical feature of β‐thalassemia intermediate with mild anemia and splenomegaly (Hb: 7.4 g/dl; A2:4.2% and HbF: 51%). The Hb Siirt variant has been previously described from Kurdish patient as a silent Hb variant (Bianco et al., 1997). A recent study reported a possible small contribution to the instability of helix β of hemoglobin β (Cappabianca et al., 2017).

We compared the frequency of mutations detected in this study with other data reported for the neighboring countries such as Algeria, Tunisia, and Senegal. The β^S^ (HBB:c.20A>T), and –α‐3.7 (g.34164_37967del3804) have been previously reported in these three countries with a high frequency in Senegal with 8%–10% for the β^S^ and 21% for –α‐3.7 (Gueye Tall et al., 2017; OMS, 2006). Two previously study reported the high frequency of β^S^ in Mauritanian population with 5.71% (Veten et al., 2012) and 8.71% (Deyde et al., 2002). In Tunisia and in Algeria, the frequency of β^S^ was, respectively, 1.89% (Fattoum, 2006; Fattoum et al., 2004) and 0.8%–3.5% (Boudrahem‐Addour et al., 2009; Mesbah‐Amroun et al., 2008). We noted that cd24 T>A (HBB:c.75T>A) and Hb Siirt (HBB:c.83C>G) seem to be specific of Mauritanian population.

The genotypic study showed eight different genotypes in our cohort. The analysis of the hematological indices associated with these genotypes indicates that the combination of β^S^/β^−thal^ increases levels of hematocrite and HbA1, and decreases levels of HbF and HbS as compared to the **β** ^**S**^ **/β** ^**S**^. Whereas, **β** ^**S**^ **/β** ^**S**^ **, αα/−−** genotype decreases MCV, hematocryte, and HbA2 more than the **β** ^**S**^ **/β** ^**S**^ genotype. These findings are in agreement with the fact that coinheritance of β^S^/β^Thal^ and β^S^/^α−thal^ modulates the severity of the disease (Saleh‐Gohari & Mohammadi‐Anaie, 2012; Valavi et al., 2010).

The molecular study of eight SNP within the β‐globin gene cluster showed that six different haplotypes among the SCD subjects with the Senegalese haplotype as the most prevalent (66.7%), followed by Benin, Arabo‐Indian, Bantu and two atypical haplotypes. These results are agreement with those found in a previous Mauritanian study carried (Veten et al., 2012) and in disagreement with those found in the Tunisian population whose the most predominant haplotype is the Benin (Moumni et al., 2011). These different haplotypes observed here reveal that was a multiple origin of β^S^ mutation in the Mauritanian population as well as the high prevalence of the Senegalese haplotype appears to be due to the gene flow of β^S^‐gene from the Senegal to the Mauritanian population.

The analysis of clinical and hematological data showed that the Senegalese haplotype presented a less severe phenotype compared to Benin, Bantu, and Arabo‐Indian haplotypes. This finding would indicate the existence of clinical variability associated with β^S^‐globin haplotypes described in our cohort. However, this association was not significant. Further studies, including a large sample size, are required to confirm our findings.

In fact, it has been previously reported that the Senegal and Arabo–Indian haplotypes are generally associated with a mild course in SCD and the individuals with the Bantu haplotype appear to have a more severe disease (Nagel & Fleming, 1992; Padmos et al., 1991).

## 5 CONCLUSION

Our results showed a high prevalence of Hemoglobinopathies patients in Bank Senegal River in the South‐West of the country. Two Mauritanian ethnic groups, the *Heratin* and the *Pular*, were the most affected.

The molecular study showed the presence of different Hemoglobinopathies in Mauritania, with SCD and β‐thalassemia as the most common hemoglobin disorders, and with an admixture of the African, Mediterranean, and Asian mutations on the Mauritanian population. The β^S^ mutation was the most common one 68.75%. Six different haplotypes have been described among the SCD subjects with the Senegal haplotype as the most prevalent. This genetic variability could be due to the geographical origin and to the multi‐ethnicity of the Mauritanian population.

Our results enrich the epidemiological data in our population and help to provide an efficient genetic counseling among families. Unfortunately, our study is not without limitations and these should be mentioned: First, the sample size is limited. Second, our patients only come from two hospital centers in Nouakchott, a multicenter study is necessary to increase the number of patients and reach all Mauritanian cities.

## AUTHOR CONTRIBUTIONS

Mr. Taher Mahmoud: The data collection, practice, data analysis, and manuscript preparation. Dr. Chaima Sahli: Experimental protocol, data analysis, and manuscript preparation. Dr. Sondess Hadj Fredj: manuscript revision Dr. Yessine Amri: Experimental protocol and data analysis. Mrs. Rim Othmani: Experimental protocol and data analysis. Pr. Taieb Messaoud: Planning, conduct, and data analysis. Dr. Sidi M. Ghaber: Conceptualization and design analysis. Dr.Ekhtelbenina Zein. The doctor providing the clinical data of patients.

## CONFLICT OF INTEREST

The authors report no conflict of interest.

## Data Availability

The data that support the findings of this study are available on request from the corresponding author. The data are not publicly available due to privacy or ethical restrictions.

## APPENDIX A

### TABLE A1 Demographic, hematological, and clinical data of the sample study

**Table:**

Patients	Age (an)	Sex	Ethnic group	Origin	Hb g/dl	MCV (fl)	MCH Pg	Ht (%)	A (%)	(A2%)	F (%)	S (%)	C (%)	Clinical signs	Genotypes
1	13	F	Heratin	Gorgol	8.1	62.4	21.5	23.49	45	4.4	17.4			MA and BT	β^−Thal^/β^−Thal^
2	3	F	Heratin	Gorgol	10.2	68.8	15	19.10	77.9	5.7	5.4			MA	β^A^/β^−Thal^
3	4	M	Heratin	Gorgol	5.4	68.8	23.8	15.64	68.4	3.3	11			SA and SHM	β^A^/β^−Thal^
4	14	M	Heratin	Noukchott	4.9	76.2	27.5	7.30	28.3	7.5	64.2			AS, BT, and SHM	β^−Thal^/β^−Thal^
5	6	M	Pular	Trarza	9.5	67	21.9	29	35.6	2	4.8	45.9		MA and VOC	β^S^/β^−Thal^
6	16	M	Heratin	–	7.4	103	32.9	22.6	26	4.2	51.1			MA and SHM	β^A^/β^−Thal^ (Hb Siirt)
7	15	M	Heratin	Assaba	11	68	20.7	36	21.7	3.1	1.5	70.4		MA and BT	β^S^/β^−Thal^
8	7	F		–	12	68	20.3	–	94.3	5.7				MA	β^A^/β^−Thal^
9	29	M	Soninke	–	13.4	67.8	21.4	–	89	7.4	3.6				β^A^/β^−Thal^
10	19	M	Pular	–	8.3	70	14	–	61.9	6	32.9			MA	β^A^/β^−Thal^
11	5	M	Soninke	GuidiMaka	5.4	84	29.8	15	2.2	1.7	16.8	75		SA and SHM	β^S^/β^S^, αα/α‐^Thal^
12	3	F	Pular	Gorgol	8.4	83	27.1	26	0	3	26.7	70.3		AM, BT, and VOC	β^S^/β^S^
13	10	F	Pular	Brakna	8.3	61.2	21.7	11	2.3	1.6	8.3	83.6		AM, BT, VOC, and SHM	β^S^/β^S^
14	13	F	Pular	Gorgol	8.6	86.8	29.7	25	21.8	2.7	6	62.5		MA and BT	β^S^/β^S^
15	11	F	Heratin	Gorgol	5.8	88.7	28.2	18.20	0	3	23.4	73.6		SA, BT, and VOC	β^S^/β^S^
16	5	F	Pular	H.Chargui	7.3	72.8	23.2	23.02	53.3	2.6	8.4	35.7		MA and SHM	β^A^/β^S^
17	7	M	Heratin	Nouakchott	7.4	61	23.7	–	0	2	0	98		MA	β^S^/β^S^, αα/α^Thal^
18	8	M	Pular	Nouakchott	7.7	78	26	14	0	2.4	30.8	66.8		MA and VOC	β^S^/β^S^
19	10	M	Pular	Trarza	10.3		33.4	–	0	3.2	12.1	84.7		MA	β^S^/β^S^
20	15	M	Heratin	Brakna	8.1	96	31.2	25	3.1	1	10.9	76		MA and VOC	β^S^/β^S^
21	15	M	Bidan	Nouakchott	11	71.3	23.7	–	50	2		48		MA	β^A^/β^S^
22	14	M	Heratin	Nouakchott	8.2	93	33	23	0	3.4	6.8	89.8		MA and VOC	β^S^/β^S^
23	9	M	Heratin	Nouakchott	3.3	70	18.9	12	4.9	5.2	0	89.9		SA	β^S^/β^S^
24	9	M	Heratin	Trarza	4.3	76	19.6	–	0	4.3	3.4	92.3		SA, BT, and SHM	β^S^/β^S^
25	4	M	Heratin	Gorgol	9	82	30	–	0	2.9	20.9	76.2		MA, BT, and VOC	β^S^/β^S^
26	15	F	Pular	Trarza				–	0	3.1	6.8	90.1		MA, BT, and VOC	β^S^/β^S^
27	6	F	Pular	Gorgol	7.3	88.6	31	20.80	13.8	1.3	5.5	65.9		MA, BT, and VOC	β^S^/β^S^
28	8	M	Pular	Brakna	10.3	61	19.1	23	47.1	1.2	0.7	33.8		MA	β^A^/β^S^, αα/α‐^Thal^
29	11	M	Heratin	Assaba	11.3	75	25	33	2.5	1.9	3.1	41.7	40.8	MA	β^S^/β^C^
30	8	M	Bidan	Tris‐Zemour	10	102	33.2	30.10	2.7	0.3	26.2	62		MA and VOC.	β^S^/β^S^
31	5	F	Bidan	Adrar	9.5	96.6	33.6	27	0	2.4	18.4	79.2		MA and VOC.	β^S^/β^S^
32			Heratin	Nouadhibou				–	84	2.8	0.8				β^A^/β‐^Thal^
33	12	F	Pular	Brakna	2.9	73	22.5	–	0.3	11	19	69.6		SA and BT	β^S^/β^S^
34	13	M	Pular	Trarza	6.8	89	29.5	18.90	12.3	3.2	5.1	79.4		SA, BT, and VOC	β^S^/β^S^
35	1	M	Pular	Brakna	5.4	92	28.5	17	0	2.8	14.3	82		SA, BT, and VOC	β^S^/β^S^
36	22	F	Wolef	Nouakchott		72.8	24	32	60.2	3.1	0	36.7			β^A^/β^S^
37	11	F	Heratin	Gorgol	6.8	89	28.5	21	0	3.9	5.7	90.4		SA, BT, and VOC	β^S^/β^S^
38	11	M	Bidan	Adrar	6.4	96	31.4	–	0	3	7.9	89.1		SA, BT, andVOC	β^S^/β^S^
39	8	F	Heratin	Trarza	7.7	82.3	26	–	0	3.1	12.2	84.7		AM	β^S^/β^S^
40	14	M	Heratin	Trarza	8.3	79.4	29.7	–	0	3.2	7.8	89		AM	β^S^/β^S^

Abbreviations: F, female; M, male; Origin, Mauritanian cities; Hb, hemoglobin; MCH, mean corpuscular hemoglobin; MCV, mean corpuscular volume; Ht, hematocrite; A, A2, F, S, and C, hemoglobin fractions; MA, moderate anemia; SA, severe anemia; BT, blood transfusion. VOC, vaso‐occlusive crises, SHM, Spleno‐Hepato‐Megaly; −, unidentified origin.
